# Target Bioconjugation of Protein Through Chemical, Molecular Dynamics, and Artificial Intelligence Approaches

**DOI:** 10.3390/metabo14120668

**Published:** 2024-12-02

**Authors:** Sk Jahir Abbas, Sabina Yesmin, Sandeepa K. Vittala, Nayim Sepay, Fangfang Xia, Sk Imran Ali, Wei-Chun Chang, Yao-Ching Hung, Wen-Lung Ma

**Affiliations:** 1 Graduate Institute of Biomedical Sciences, China Medical University, Taichung 40402, Taiwan; 2 Department of Obstetrics and Gynecology, Asia University Hospital, Taichung 41354, Taiwan; 3 Institute of Chemistry, Academia Sinica, Taipei 115201, Taiwan; 4 Department of Physics, National Dong Hwa University, Hualien 97401, Taiwan; 5 Leiden Institute of Chemistry, Leiden University, 2300 RA Leiden, The Netherlands; 6 Department of Chemistry, Lady Brabourne College, Kolkata 700017, India; 7 Department of Epigenetics and Molecular Carcinogenesis, University of Texas MD Anderson Cancer Centre, Houston, TX 77030, USA; 8 Department of Chemistry, University of Kalyani, Kalyani 741235, India; 9 Ph.D. Program for Health Science and Industry, China Medical University, Taichung 40402, Taiwan; 10 Department of Medical Research, Department of Obstetrics and Gynecology, China Medical University Hospital, Taichung 40402, Taiwan

**Keywords:** bioconjugation, protein modification, computational method

## Abstract

Covalent modification of proteins at specific, predetermined sites is essential for advancing biological and biopharmaceutical applications. Site-selective labeling techniques for protein modification allow us to effectively track biological function, intracellular dynamics, and localization. Despite numerous reports on modifying target proteins with functional chemical probes, unique organic reactions that achieve site-selective integration without compromising native functional properties remain a significant challenge. In this review, we delve into site-selective protein modification using synthetic probes, highlighting both chemical and computational methodologies for chemo- and regioselective modifications of naturally occurring amino acids, as well as proximity-driven protein-selective chemical modifications. We also underline recent traceless affinity labeling strategies that involve exchange/cleavage reactions and catalyst tethering modifications. The rapid development of computational infrastructure and methods has made the bioconjugation of proteins more accessible, enabling precise predictions of structural changes due to protein modifications. Hence, we discuss bioconjugational computational approaches, including molecular dynamics and artificial intelligence, underscoring their potential applications in enhancing our understanding of cellular biology and addressing current challenges in the field.

## 1. Introduction

Proteins are essential functional biomolecules among various cellular components, playing vital roles both within and outside living cells. Due to their unique structures and chemical reactivity_,_ they have garnered significant attention ranging from therapeutics to biomaterial applications, such as 3D cell culture and disease modeling [1,2,3]. Consequently, increased efforts have been devoted to protein bioconjugation, aiming in terms of both material applications to regulate their spatial–temporal interactions and biomedical applications related to signal transduction and protein localization. To achieve these applications, site-selective modification of proteins was employed without compromising their inherent structure and function. Using the available chemistry toolbox, chemical biologists have developed high-throughput protein modification techniques with improved bioavailability and targeted delivery to overcome conjugation challenges. Given the fragility and size of proteins, important prerequisites of site-specific protein modification include: (1) the maintenance of mild aqueous reaction conditions; (2) avoidance of cross-reactions with unprotected groups; and (3) selecting reactions that proceed relatively quickly and at low concentrations. To achieve these modifications, various strategies were employed by taking advantage of the nucleophilicity, pKa, redox potential, and reaction-based modifications using maleimides, N-hydroxysuccinimide (NHS) esters, and α-halocarbonyls to perform modifications. Most of the reported reactions, such as Michael addition, activated ester amidation, and reductive amination, have existing advantages and disadvantages in improving the reaction rate and achieving product formation. In addition, the specificity of site-selective protein modification can be improved by utilizing reactive small-molecule reagents and enzymes and by harnessing protein translational processes [4,5].

**Figure 1:**
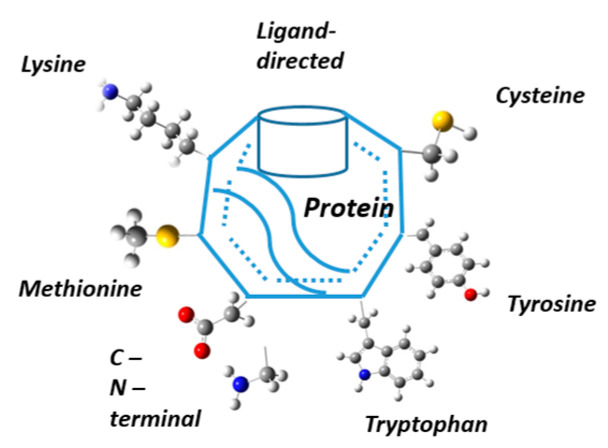
Schematic representation of the protein modification sites. The C-terminal, N-terminal, and various amino acid including lysine, cysteine, tyrosine, tryptophan and methionine, represent the reactive modification site. Colours of the various atoms within the molecules as follows, Hydrogen = White, Carbon = Grey, Nitrogen = Blue, Oxygen = Red, Sulphur = Yellow.

These site-selective protein modification strategies are specifically on the basis of structural and functional sites in proteins. The resultant modification provides various applications in the protein research, such as studying receptor trafficking mechanisms, investigating protein localization, and detecting signal transduction [3,6,7,8,9,10,11,12]. To better understand the biological events, an important strategy is the incorporation of post-translational modification (or labeling) to the target protein in a site-specific manner. The modification of functional properties is a vital tool for interrogating and intervening in biological systems. It is of particular interest in the fields of biotechnology, such as protein-based biosensors and biomaterials studies [13,14,15,16,17]. Additionally, in the field of biophysical measurement, these modifications are important for applications like diagnostics and protein−protein interactions [18,19,20]. In the field of medicine, protein-based therapeutics are used for cancer treatment and the enhancement of individual improvement of protein properties [21,22,23,24]. Although this procedure offers significant advantages, there are still many limitations to be addressed to improve complementary reactions. In this context, numerous studies have explored different strategies and challenges [5,25,26,27,28]. Here, we focused on the progress of current state-of-the-art methods, covering recent examples of modification methodologies with comprehensive information on single-site modification of native proteins (Figure 1). These include (i) naturally occurring amino acids (AA) via chemo- and regioselective approaches and (ii) protein-selective, proximity-driven methods.

We also discuss the use of computational methods, such as (i) molecular dynamics and (ii) artificial intelligence, to identify amino acids or the site where bioconjugation is feasible and to evaluate its effect on the stability and behavior of the protein in its microenvironment. Additionally, we covered biomedical applications including protein therapeutics, drug delivery, molecular diagnostics, protein stabilization, and tissue engineering.

## 2. Modification of Naturally Occurring Amino Acids Through Chemo and Regioselective Approaches

This section highlights a single-site modification of specific or native proteins. The key approach involves chemo- and regioselective modification in a site-selective manner, addressing several important aspects such as labeling-site information, chemical topographies, and the development of bioconjugates or conjugates at specific amino acid groups. Examples have been reported based on the tuning capability of the external electrophiles to target specific nucleophiles of amino acids in complex environments. This may also include other non-target proteins and reactive amino acids.

**Scheme 1:**
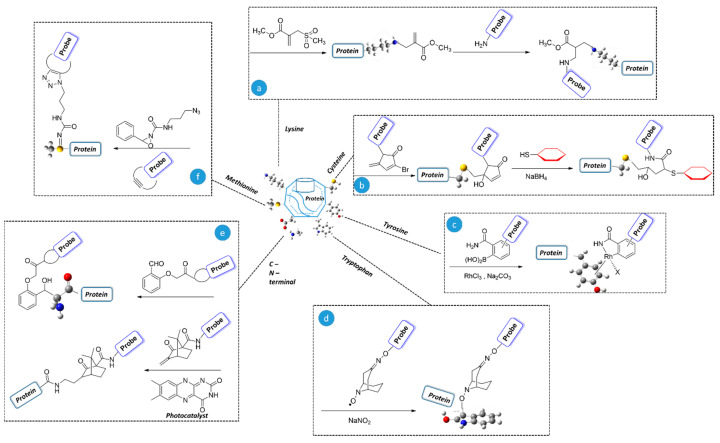
Chemical modification of native protein amino acids and probes indicates the chemical handles or functionalities of the respective molecules. Chemical modifications include the following: (**a**) lysine on native proteins, with the reaction benefits from a chair-like H-bonded transition state and aza-Michael addition reaction [40]; (**b**) cysteine modified with 3-bromo-5-methylene pyrrolones (3Br-5MPs) followed by a thiol conjugation reaction [45]; (**c**) tyrosine modified with boronic acid and rhodium salt, followed by η6-arene Rh complexes [55]; (**d**) tryptophan modified with N-oxyl radical, 9-azabicyclo[3.3.1]nonane-3-one-N-oxyl (keto-ABNO) [51]; (**e**) C- and N-modifications achieved through under visible-light catalyzed reactions and transfer of amino alcohol under physiological conditions [63]; and (**f**) methionine modified with oxaziridine reagent [54].

In protein conjugation, the commonly targeted side chains of amino acid residues are the ε-amino group of lysine (Lys) and the sulfhydryl group of cysteine (Cys). Due to their higher relative abundance on protein surfaces (5.9% for Lys in human proteins and 2.3% for Cys in the genome), biochemists considered them to be prime targets for modification, owing to their nucleophilicity [29,30,31,32,33]. Additionally, implementing reactive chemistry toward organic functional groups has been reported. Achieving faster reactivity with higher selectivity requires optimal conditions, such as specific pH and temperature, along with the use of appropriate reagents [32,33,34,35,36,37,38,39]. Bernardes and colleagues addressed sulfonyl acrylate reagents that facilitated single-site-selective modification of Lys (Scheme 1a) under favorable conditions (~100% yield, basic pH, 1–2 h, and ambient temperature: 25–37 °C). The excellent selectivity towards particular amino acids is due to intermolecular transient hydrogen bonding between the reagent and reactant ε-amino group of Lys, leading to further development [40]. In addition, the literature reports that under catalyst-free conditions, it can drive N-terminal modification [41]. Davis et al. introduced two-step Cys modification through the reactive intermediate dehydroalanine (Dha, which has a versatile reactivity profile), followed by Michael addition with O-mesitylenesulfonyl-hydroxylamine (MSH) and allylthiol nucleophiles, respectively. Meanwhile, the same group also introduced bis-amide reagent instead of MSH, which tuned the elimination strategy under favorable conditions (pH 8.0 and 37 °C) [42,43,44]. Zhou et al. developed multi-functionalized Cys-specific protein modification with 3-bromo-5-methylene pyrrolones (3Br-5MPs). The modified conjugates can further undergo an additional reaction with another thiol (~95% yield, pH 7.5, 1 h, and 37 °C) followed by reduction under mild conditions to generate biologically active and stable conjugates (Scheme 1b) [45]. Li et al. reported that Cys labeling directed toward Lys site-selective modification [46].

Apart from the Lys and Cys, other naturally occurring amino acid residues are also used for selective bioconjugation. Among the low-abundance canonical amino acid residues, tyrosine (Tyr), tryptophan (Trp), and methionine (Met) are particularly interesting candidates for chemo-selective modification, in which Tyr-Trp and Met modifications respond through ring electron and redox sites, respectively. Due to the unique chemical properties, a wide variety of applications have been reported [47,48,49,50,51,52,53,54]. Ball and co-workers modified a Tyr side chain with boronic acid in the presence of a transition-metal complex of rhodium salt, involving organometallic linkage (η6-arene Rh complexes, pH 9.4, overnight, and room temperature) and metastability/reversibility maintained with dithiothreitol (DTT) or H_2_O_2_ (Scheme 1c) [55]. Tyr-selective protein modification with water-soluble iminoxyl radicals provides unique opportunities in this field [56].

These results offer valuable insight for further exploration of innovative, precise interactions. Kanai and co-workers documented a chemo-selective Trp modification using the transition metal-free organo-radical reagent N-oxyl radical, 9-azabicyclo [3.3.1]nonane-3-one-N-oxyl (keto-ABNO) to achieve a highly homogeneous product (Scheme 1d). However, this approach resulted in low conversion yields (11–64% pH 7.4, at 30 min and room temperature) due to the need for an acidic condition. To overcome this drawback, a metal-mediated, alternative efficient method has been reported [51]. Davis’s group introduced another trifluoro-methylation radical reagent for 19F-NMR probe protein conformational analysis [57].

The redox-activated chemical tagging (ReACT) channel has been reported for Met bioconjugation, using oxaziridine reagents to form sulfimide products (conversion > 95%, pH 7.4, 10 min, and room temperature) through selective oxidation (Scheme 1f) [54]. Another comparable hypervalent iodine reagent was introduced by Taylor et al. for the incorporation of acyl groups [58]. The N-terminal amino and C-terminal carboxylates in proteins contribute unique properties for homogeneous conjugation. Numerous classical approaches based on modification and their applications have also been demonstrated [30,59,60,61,62]. Purushottam et al. reported glycine-specific N-terminus labeling (Scheme 1e, top) through translational of amino-alcohol under physiological conditions (conversion 52% Melittin, pH 7.8, 48 h, 20 °C) [63]. MacMillan and co-workers addressed C-termini conjugation under visible-light catalyzed decarboxylative alkylation with Michael acceptor through single-electron transfer (conversion 31–66%, pH 3.5, 8 h, and room temperature) (Scheme 1e, bottom). The oxidation potential exhibited by C-termini plays an important role in potential research on other residues [64].

Additionally, chemo-enzymatic site-specific protein modification was reported using the OaAEP1 enzyme. Tang et al. recently described OaAEP1-C247A-aa55-351 as a potential candidate for protein engineering. Ma et al. reported OaAEP1 mediate C-terminal site-specific protein immobilization on nanoparticles [65,66].

**Figure 2:**
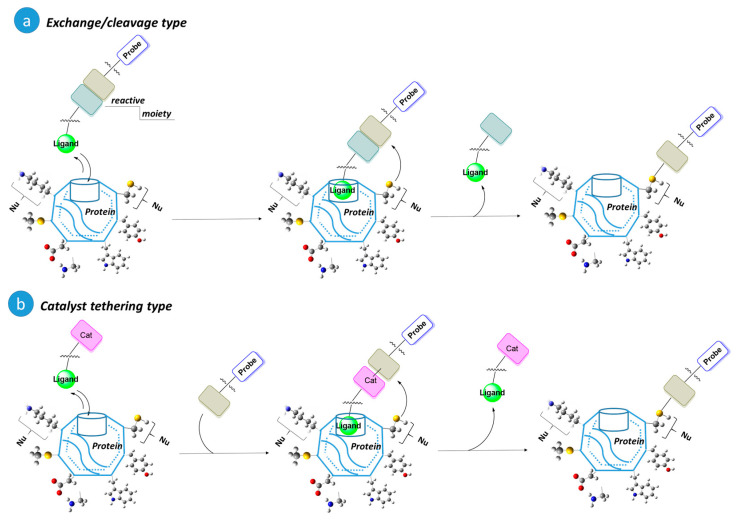
Affinity-based protein labeling: (**a**) the exchange/cleavage reaction type; and (**b**) the catalyst tethering type.

Notably, residue-specific modifications discussed above have certain limitations regarding less abundant, reactive, nucleophilicity amino acids. These issues can be explained through protein-selective proximity-driven labeling, which emphasizes molecular shape and the local environment rather than reactivity. Consequently, the focus is shifting toward a series of traceless affinity labeling techniques and affinity-guided catalysts, which involve exchange/cleavage and catalyst tethering, respectively (Figure 2) [8,28,67,68,69]. To address these challenges and enhance the effectiveness of protein modifications, researchers are increasingly exploring innovative strategies such as protein-selective proximity-driven chemical modifications.

## 3. Protein-Selective Proximity-Driven Chemical Modification

Site-specific and target-selective protein modification are achieved through affinity-based chemical labeling of endogenous proteins, specifically native proteins. The demand for these proteins is increasing due to their native habitats, minimal or no perturbation of cellular functions, and the ability to monitor activity in real time. The application in the living systems remains insufficient [70,71]. These new technologies can be categorized into three different steps: (i) identification of the ligand fragment labeling reagent (indicating probe) tethered to the catalyst; (ii) attachment of the target protein through covalent activation and adding on of the covalent probe; (iii) simultaneous cleavage or subsequent exchange of the ligand fragment, resulting in dissociation of the ligand. In both cases, the key mechanism involves ligand-protein interactions through the “proximity effect”. Which facilitates the efficient incorporation of the motifs into endogenous proteins while preserving [68].

## 4. Traceless Affinity Labeling

**Figure 3:**
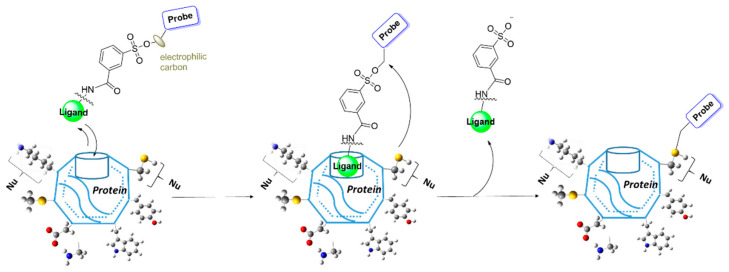
The reaction scheme of ligand-directed tosyl chemistry; Nu denotes the nucleophilic residue on the protein surface.

Affinity-based labeling is distinguished into three categories: post-photoaffinity labeling, post-affinity labelling, and ligand-directed tosyl (LDT) chemistry [72,73,74,75,76,77,78]. In this section, we emphasize the progress and challenges of specific target-selective ligand-directed (LD) application. One of the pioneering groups in this field, Hamachi et al. introduced a new labeling linker, phenyl sulfonate (tosylate). This linker plays an important role in the binding of the ligand to the reactive group (probe) and also serves as a good leaving group when a nucleophilic amino acid residue on the protein surface accelerates the SN^2^-type reaction. Following chemoselectivity intermolecular steps, the probe is transferred to the amino acid moiety while simultaneously cleaving the ligand fragment without disturbing protein activity (Figure 3) [19].

Building upon the concept of affinity-based labeling, various classifications and advancements have emerged in this field, particularly focusing on LD strategies. Phenyl sulfonate chemistry has been employed across a broad range of proteins in various crude environments, such as live cells, tissues, cell lysates, and red blood cells in mice. The LD chemistry allows for the attachment of selective chemical probes (e.g., fluorophores, affinity-tag biotin, and the 19F-NMR probes) onto endogenous proteins in diverse biological settings, from human red blood cells (RBCs) to living mice. Furthermore, FK506-binding protein 12 (FKBP12) highlights essential properties of linker rigidity, length, reaction kinetics, and applicability in living cells. Results revealed a slow labeling rate with incubation times spanning hours to days, resulting in yields that were often not significant [69,77,79,80,81,82].

**Figure 4:**
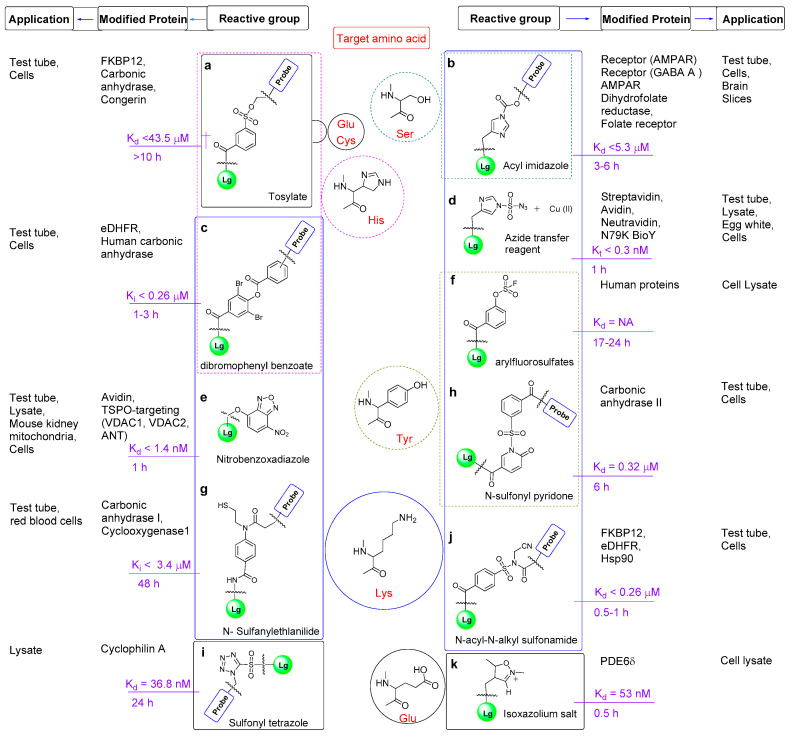
Schematic representation and summary of ligand-directed chemistry; K_d_, K_t_, and K_i_ denoted binding affinity. Reactive group of each examples representation (**a**) Tosylate; (**b**) Acyl imidazole; (**c**) Dibromophenyl benzoate; (**d**) Azide transfer reagent; (**e**) Nitrobenzoxadiazole; (**f**) Arylfluorosulfates; (**g**) N-Sulfanylethlanilide; (**h**) N-sulfonyl pyridone; (**i**) Sulfonyl tetrazole; (**j**) N-acyl-N-alkyl sulfonamide; and (**k**) Isoxazolium salt.

To address these shortcomings, Hamachi et al. expanded the range of cleavable electrophiles using the ligand-directed acyl imidazole (LDAI) motif, which exhibited a 12-fold greater efficiency than LDT. Due to the rate-determining intermolecular step, the reactive alkyloxyacyl imidazole facilitates rapid and efficient labeling of protein surfaces [83]. On the other hand, labeling efficiency also depends on the specific amino acid preferences (Figure 4a), such as Lys, serine (Ser), threonine (Thr), and Tyr, which demonstrate precise modification on the Lys residue, an area where LDT chemistry fails to provide a satisfactory explanation. Despite this drawback, LDAI chemistry positively impacts numerous nucleophilic amino acids including histidine (His), Tyr, glutamine (Glu), aspartic acid (Asp), and Cys residues [19]. Additionally, LDAI chemistry offers biological applications significantly, including pulse–chase analysis of target proteins. Moreover, it provides photo-control over enzymatic activity and specific imaging of endogenous neurotransmitter receptors. However, the acyl imidazole group is more susceptible to degradation by enzymes (e.g., esterases and proteases), limiting the applicability of this reagent inside cells [84,85,86,87,88]. To mitigate enzymatic degradation, a steric hindrance approach has been introduced using ligand-directed dibromophenyl benzoate (LDBB) reagent. The reactive group scaffold of LDBB reagents is not a good enzyme substrate for enzymes; allowing for tuned reactivity and stability and rapid intracellular protein labeling with faster kinetics is enabled [89].

Another LD strategy explored is N-sulfonyl pyridone (LDSP) chemistry, which proves useful for both intracellular and cell surface membrane proteins (Figure 4h). Additionally, it possesses unique properties under live cell conditions, allowing for the direct conversion of a native protein to a FRET-based biosensor [90]. Another case of outstanding LD chemistry was reported using LD N-acyl-N-alkyl sulfonamide (LDNASA) (Figure 4j), which demonstrated the fastest reaction kinetics among all LD scaffolds (LDT, LDAI, LDBB) [91]. Its second-order rate constant reaches ∼10^4^ M^–1^ s^−1^; nearly comparable for enzymatic labeling and bio-orthogonal reactions of inverse-electron demand Diels-Alder (IEDDA), exhibiting sufficient orthogonality for enzymatic degradation due to its unique structural scaffold [92].

Furthermore, a kinetic study supports the affinity of the ligand with sub-micromolar order for faster reaction chemistry. The effective molarity of LDNASA-based model proteins FKBP12 and Hsp90s allows for remarkably rapid and selective labeling. An important feature of LDNASA provides insight into off-target labelling and methods for improving target-selectivity [93,94]. Another report also documented LDNASA-mediated Hsp90 labeling in living cells [95]. Affinity labeling modifications of Lys residue have been extended using the smaller molecular unit of nitrobenzoxadiazole (NBD) derivatives (such as alkoxy and alkylamine substitution of NBD) and ligand-tethered diazide-transfer reagents (ligand: biotin, functioning in the presence of Cu (II) ions) [96,97]. Both of the reagents selectively modified the model protein avidin, while the first targeted TSPO-proteins (VDAC1, VDAC2, and ANT) and the second targeted streptavidin and BioY. Another intriguing reagent, N-sulfanylethylanilide, has also been documented as modifying in human carbonic anhydrase (hCA) and cyclooxygenase1 through the N-S acyl shift [98]. Clear evidence of this phenomenon is still under close investigation.

Although several groups have reported other reactive electrophile reagents, such as 5-sulfonyl tetrazole and isoxazolium salts (Figure 4i–k) [99,100,101]. Woodward’s reagent K (which resembles isoxazolium salts) was introduced for the modification of less reactive carboxylic acid amino acids Asp and Glu in the lipoprotein chaperone PDE6δ. Additionally, significant latent electrophiles, like aryl-fluorosulfates, are being explored for drug discovery strategies, although their reactivity appears slow for modifying amino acids in human proteins within living cells [102,103,104]. This method also opens potential avenues for covalent inhibition approaches. However, certain limitations arise regarding ligand derivatives that contain nucleophile groups in their structures, which can disrupt labeling reagents through intermolecular decomposition and hinder the binding of small-molecule ligands to the protein.

## 5. Affinity-Guided Catalyst

**Figure 5:**
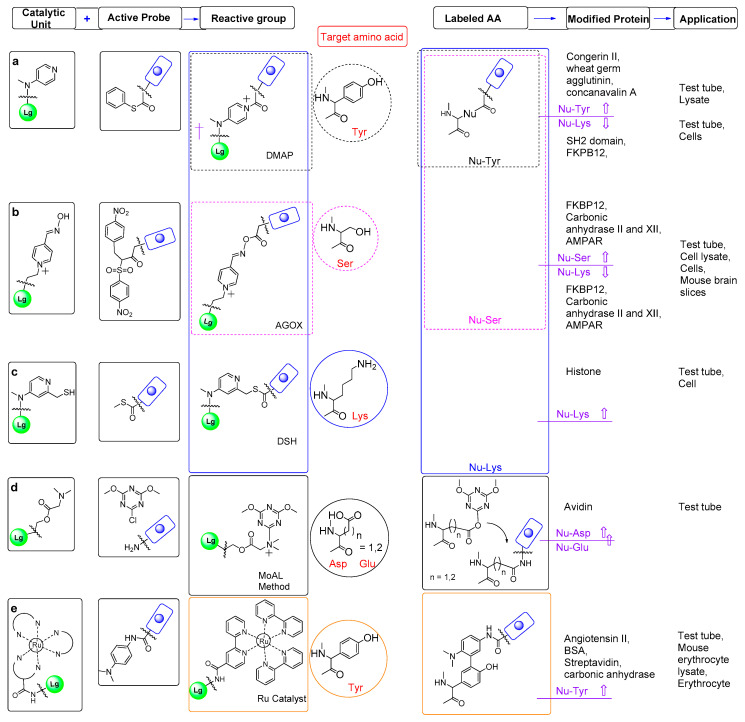
Schematic representation of affinity-guided catalyst chemistry, provide the information of activation of amino acid. Reactive group of each examples representation (**a**) DMAP; (**b**) AGOX; (**c**) DSH; (**d**) MoAL method; and (**e**) Ru Catalyst.

This class of catalyst-mediated labeling of endogenous proteins, reported by the Hamachi group, utilizes organocatalysts designed to achieve specific affinity for a target protein. This approach, known as affinity-guided DMAP (4-dimethylaminopyridine) (AGD) chemistry, promotes acyl transfer reactions activating and transferring acyl groups from the thiophenyl ester to nucleophilic amino acids, such as Lys and Tyr (Figure 5a) [105,106]. AGD chemistry has been widely applied to facilitate the submission of nucleophile-containing small-molecules, proteins, and antibodies bearing nucleophilic amino acid recognition modules [107,108,109]. Moreover, it supports traceless protein labeling across various membrane proteins in liver cells, including global modification of glycoproteins and the selective labeling of endogenous HER2 and EGFR [107,108].

Kunishima et al. introduced a three-component reaction using a ligand catalyst of biotin-conjugated dimethylglycine (DMG), the reactive module of 2-chloro-4,6-dimethoxy-1,3,5-triazine (CDMT), and a primary amine probe. Initially, reactive species form between DMG and CDMT, followed by the formation and generation of an activated ester (4,6-dimethoxy-1,3,5-triazinol esters) with the protein residue Asp or Glu, leading to an acylation reaction with the amine probes (Figure 5d). This method, known as modular affinity labeling (MoAL), expands the tool kit for protein modification [110].

To enhance DMAP catalysts, Kanai and colleagues developed a new DMAP-SH (DSH) system (Figure 5c), which boosts the activity of stable thioesters (e.g., acetyl-CoA) and enables the acyl group transfer to proximal amino residues. This approach allows for histone protein labeling in a recognition-driven manner [111,112]. Other alternative strategies include boronate-assisted hydroxamic acid (BAHA) catalyst systems, which offer bonafide bio-orthogonal reactions applicable in chemical biology [113].

The Hamachi group also explored affinity-guided oxime catalysts combining pyridinium oxime and NASA, where oxime functions as an acyl transfer agent and NASA as an acyl donor (Figure 5b). This catalyst enhances the reaction conditions (e.g., pH) and improves the bioorthogonality of the acyl donor, facilitating endogenous labeling of native-form proteins, such as membrane protein, carbonic anhydrase XII, and neurotransmitter receptors, e.g., AMPA receptors in mouse brain tissue [114]. Another report introduced modified NASA structures, such as ArNASAs, along with derivative libraries that define favorable electrophiles that have been defined for use in lysine-targeted covalent inhibition of ibrutinib-resistant BTK mutants [115]. Additionally, Nakamura et al. developed photoredox chemistry using a tethered transition metal ligand catalyst (Figure 5e), demonstrating feasibility in live-cell environments and reactivity toward residues in a fluorophore-substituted dimethylaniline derivative [116,117].

## 6. Molecular Dynamics-Guided Labeling

In the preceding sections, we have thoroughly explored the methods and chemicals involved in bioconjugation. It’s important to recognize that the attachment of a chemical to a protein can significantly impact its native structure and function. Additionally, proteins often possess multiple target functional groups, each in distinct environments, raising the question of which group will react in a given scenario. With the advancements in computational facilities, we are now well-equipped to tackle these complex challenges related to bioconjugation. Numerous computational studies have recently emerged, focusing on the development of innovative bioconjugation methods, elucidating the effects on protein structure, and discovering new drug candidates. These advancements are integral to our discussion.

The enhancement of physiologically significant biologics through covalent modification of proteins with polymers has gained prominence in recent decades due to a shift from small-molecule drug therapies to protein-based biopharmaceuticals. However, biologics often suffer from ex-vivo degradation, aggregation, immune response, and rapid renal filtration, which can limit their practical use in medical applications. A primary reason for these limitations is the lack of covalent attachment between biological macromolecules and synthetic polymer compounds. Bioconjugation offers a promising solution approach to overcoming these challenges by improving the physicochemical and pharmacokinetic properties of the active ingredients, thus enhancing the stability and efficacy of protein-based therapies [118,119].

Simulating protein–polymer bioconjugates, such as PEGylated (or pegylation is a process binding of polyethylene glycol to a molecule), is an essential step toward optimizing these therapeutic approaches. However, this simulation process faces specific challenges due to the limited availability of force field parameters and starting coordinates for polymers. Unlike proteins, polymer chains and their linkers are not typically covered by standard force fields, requiring separate deviations of parameters. Various force fields, including CHARMM, AMBER, GROMOS, NAMD, and OPLS-AA, provide methods for deriving or generating parameters for polymers and linkers while ensuring compatibility of partial atomic changes with the chosen force field set [120,121,122,123,124].

**Figure 6:**
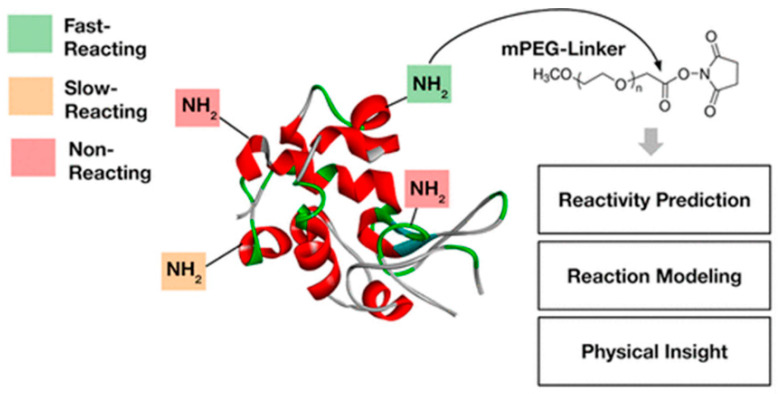
Overview of the structure-dependent reactivity kinetic model which can provide site-modification predictions and simulate PEGylation reactions. Adapted from an open access journal [125].

For many years, the field of protein PEGylation has lacked a comprehensive model to identify preferred PEGylation sites and accurately predict conjugation rates and outcomes. Carmali addressed this gap by developing a structure-dependent reactivity model that quantitatively predicts Lys reactivity and considers the polyethylene glycol (PEG) shielding distance to determine the reactivity sequence. This model facilitates the rapid optimization of conjugate yield and specificity, thereby streamlining the development of PEGylated proteins in an efficient and data-driven manner. It accurately predicted the reactivity order of BChE and showed a strong correlation when validated with PEGylated proteins, while also being used to simulate PEGylation progress and estimate PEGmer distribution with high accuracy (Figure 6) [125].

**Figure 7:**
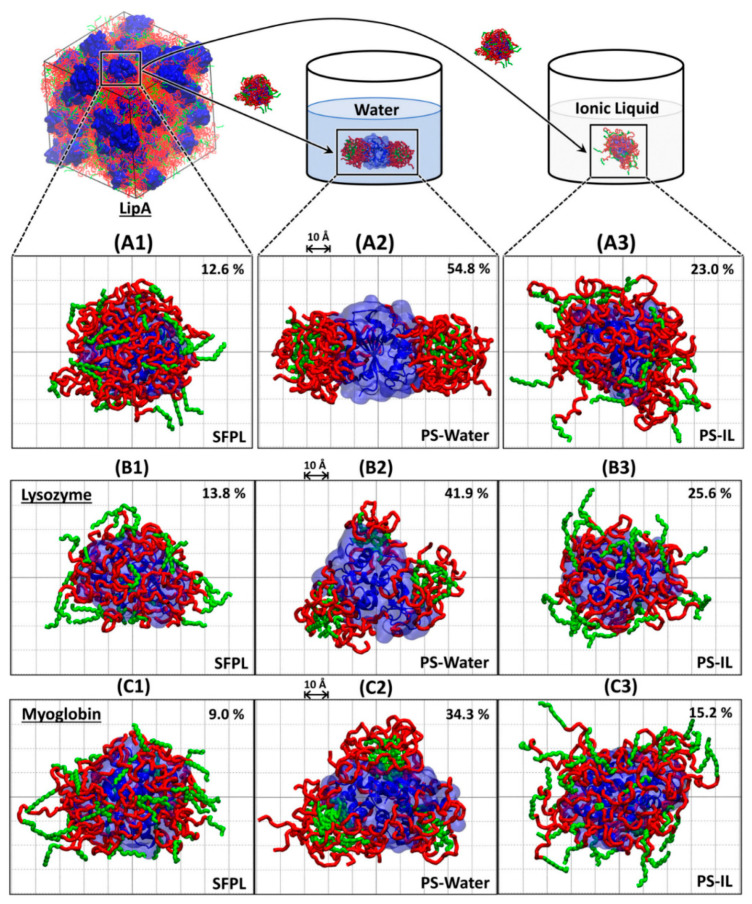
In the simulation box, there are sixty-four enzymes in the SFPL of protein with representations for the enzyme, PEG, and the alkyl parts of the surfactant represented by a blue surface, red lines, and green lines, respectively. Here, (**1**) the initial structure of one PS complex of LipA extracted from its SFPL, (**2**) the structure of the PS complex of protein in the last MD frame in a PS–water simulation, and (**3**) the structure of the PS complex of protein in the last MD frame in a PS-IL simulation. (**A**–**C**) panels show LipA, lysozyme, and myoglobin, respectively. Adapted with copyright permission from [126].

To further illustrate the benefits of protein–polymer bioconjugates, recent studies have employed molecular dynamics simulations to investigate the unique properties of protein–polymer surfactant bioconjugates in ioninc liquids (ILs). Balasubramanian and co-workers explored the factors contributing to the exeptional thermal stability, solvent tolerance, and solubility of these bioconjugates (Figure 7) [126]. Solvent-free protein liquids (SFPL), composed of protein–polymer surfactant bioconjugates, offer proteins the ability to operate at higher temperatures compared to aqueous solutions, although they exhibit high viscosity. Dissolving SFPLs in ILs with lower viscosities can significantly enhance catalytic rates, thereby enabling high-temperature catalysis while avoiding the solubility and stability issues commonly encountered with neat proteins in ILs.

Extensive atomistic molecular dynamics simulations of three proteins (LipA, lysozyme, and myoglobin) provided insights into the mechanisms behind the increased thermostability and solubility of proteins in their SFPL-IL forms. The key findings from the simulations are as follows: (i) thermal stability: the surfactant layer surrounding the proteins in SFPL-IL remains intact, ensuring higher thermal stability than in aqueous solutions; (ii) rigidity and viscosity: the combined effect of the coronal surfactant layer and the higher viscosity of IL offer greater rigidity to the proteins, leading to increased thermal stability and imroved catalytic performance; (iii) enthalpic contributions: enhanced thermostability is driven by a greater number of intra-protein hydrogen bonds in the SFPL-IL state; (iv) protection from structural distortion: the surfactant layer acts as a shield, preventing structural distortions that neat proteins often experience in Ils; (v) improved solubility: the decreased surface polarity of the protein–polymer hybrid allows better solubility in IL, supported by substantial hydrogen bonding between the surfactant and IL components.

The field of protein–polymer conjugates continues to grow with regard to therapeutic applications, addressing challenges such as synthesis, characterization, and achieving desired effectiveness. Variations in factors like modifications location, polymer density, length, shape, and interactions with the protein surface have led to a diverse range of potential conjugates, making it essential to optimize these parameters for therapeutic success.

**Figure 8:**
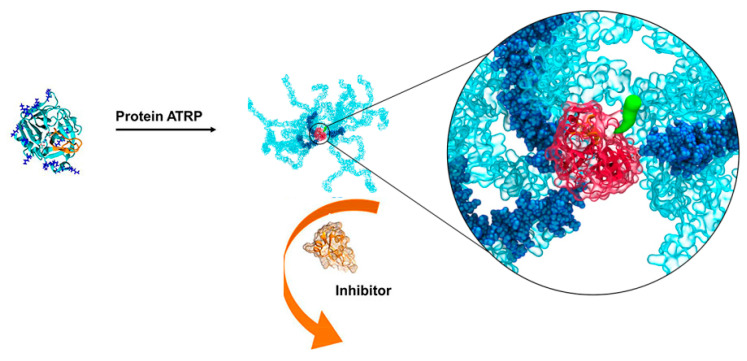
Bioconjugation of native protein with polymers inhibiting the protein–protein interactions. Adapted from open access journal [127].

Russell and his team advanced this area by utilizing molecular dynamics simulations to guide the design of protein–polymer conjugates that could effectively shield the enzyme surfaces from protein-based inhibitors (Figure 8) [127]. By combining molecular dynamics-guided synthesis of of protein–polymer conjugates via atom transfer radical polymerization (ATRP) with absolute molar masses determination using asymmetrical flow field–flow fractionation (AF4), they evaluated the functionality of molecular sieve architectures, including linear-, branched-, and comb-shaped conjugates.

The results showed that high-density comb-shaped conjugates were the most effective at completely inhibiting protein–protein interactions while retaining enzymatic activity. This high-density arrangement created tight molecular sieves compact enough to prevent inhibitor binding. The integration of AF4 with multi-angle light scattering and dynamic light scattering provided insights into the compact and spherical architecture of comb-shaped conjugates.

Further analysis revealed that these chimera structures, especially those featuring comb-shaped polymers, significantly enhanced enzyme stability and minimized protein interactions. Molecular simulations indicated that a dense nano-armor with long, comb-like polymer arms could effectively filter out large ligands, achieving complete inhibition of protein–protein interactions with a polymer composition of 99%.

These findings not only underscore the potential of molecular dynamics simulations in the rational design of protein–polymer conjugates but also open new avenues for developing therapeutic proteins by understanding the molecular basis behind their behavior.

Considering the growing interest in protein–polymer conjugates and their impact on enzyme behavior, the enhancement of enzyme catalysis through biopolymer scaffold modification represents a powerful approach. While modification of enzyme scaffolds is widely used to improve catalytic performance, predicting how the scaffold’s position will influence enzyme properties remains a significant challenge.

**Figure 9:**
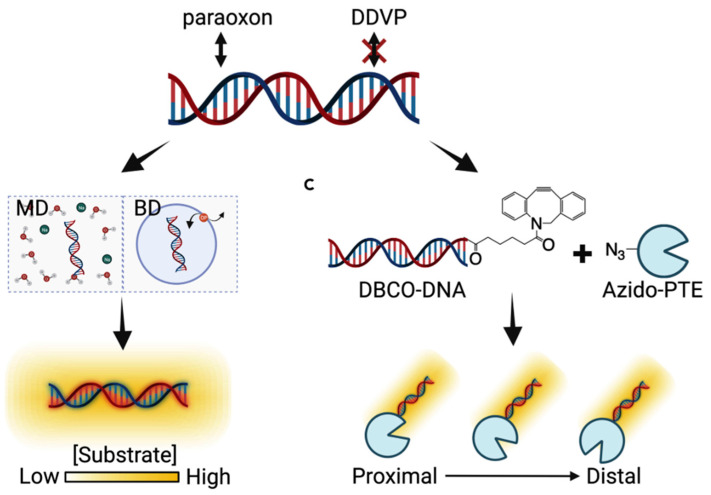
A multiscale computational and experimental approach demonstrating the influence of genetic scaffolds on enzyme kinetics at the positional level. Adapted with copyright permission from [128].

Wheeldon and colleagues addressed this by investigating phosphotriesterase (PTE) modified with a 20 bp double-stranded DNA (dsDNA), demonstrating that the conjugation position is just as crucial as the chemistry and structure of scaffold (Figure 9) [128]. Their kinetic analysis revealed that positioning scaffold next to, but not obstructing, the active site maximized the effective concentration provided by the scaffold, thereby enhancing enzyme activity.

Utilizing a molecular dynamics model that incorporated substrate concentration and the geometry of the PTE-DNA conjugate, they could accurately predict kinetic improvements across various scaffold positions. This approach highlighted the capacity of DNA, protein, and polymer scaffolds to achieve nanoscale-level precision in modifying enzyme microenvironments, thereby tailoring enzyme particles to specific size, shape, and activity requirements.

Moreover, the study demonstrated that altering the chemical and physical microenvironment of enzymes through the conjugated DNA can controllably influence enzyme kinetics. This finding paves the way for more sophisticated design strategies for enzyme-based applications, where the precise tuning of catalytic properties is needed for biotechnological and therapeutic use.

## 7. Artificial Intelligence in Protein Labeling

**Figure 10:**
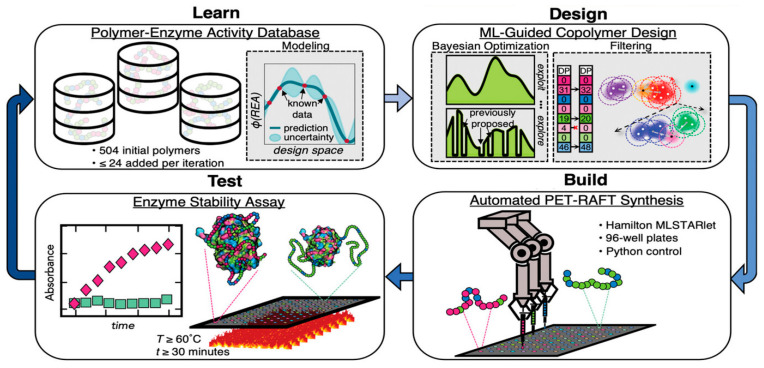
Schematic representation of closed-loop learn–design–build–test discovery process. The process involves training an enzyme-specific Gaussian process regression surrogate model to predict retained enzyme activity (REA) of a polymer–protein hybrid (PPH) based on copolymer characteristics. The next step is Bayesian optimization of copolymers to propose new copolymers, followed by automated synthesis of proposed copolymers via PET-RAFT polymerization. The synthesized copolymers are then mixed with the enzyme to form PPHs, which undergo thermal stress testing and are assessed for REA. Newly acquired data along with existing data are then used to initiate a new iteration of the process. Adapted with copyright permission from [129].

In this section, we discuss the progression from traditional methods of enzyme modification to more sophisticated, data-driven approaches for enhancing enzyme stability and activity. The development of polymer–protein hybrids using automated polymer chemistry and machine learning represents a significant leap towards creating more robust and versatile enzyme systems. The development of polymer–protein hybrids using automated polymer chemistry and machine learning has led to the discovery of hybrids with enhanced thermostability for three different enzymes. Gormley and co-workers successfully demonstrated a strategy that combines using active machine learning and automated material synthesis to design protein-stabilizing copolymers [129]. Analysis of surrogate machine learning models has revealed specific chemical features of copolymers that contribute to increased enzyme activity retention (Figure 10). This platform for polymer–protein hybrids can be extended to other proteins, copolymer chemistries, and design objectives, potentially accelerating applications in various fields. In addition, there is potential to generalize surrogate models to incorporate both protein and copolymer chemical features and use assay data with a simulation-based model to validate molecular-level mechanisms by integrating assay data with simulation-based models, optimizing the high-throughput materials discovery process. This work expands the capabilities of designing synthetic copolymers that manipulate protein activity and has broad applications in developing robust polymer–protein hybrid materials.

The study provides an overview of the surface chemistry of horseradish peroxidase, glucose oxidase, and lipase, accompanied by a schematic illustration featuring amino acid classifications and protein images rendered using visual molecular dynamics (Figure 10). It also includes the utilization of monomers for copolymer design, categorized as ionic, hydrophilic, and hydrophobic. Furthermore, the study presents a closed-loop learn–design-build–test discovery process, starting with the training of an enzyme-specific gaussian process regression surrogate model, followed by Bayesian optimization of copolymers, automated synthesis of the proposed copolymers via PET-RAFT polymerization, and the assessment of polymer–protein hybrids for enzyme activity. The iterative process used newly acquired and existing data to initiate subsequent learn–design–build–test cycles.

**Figure 11:**
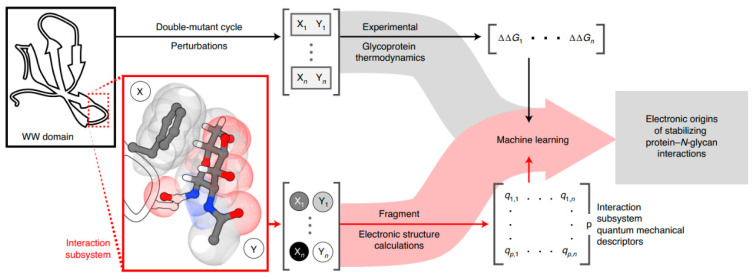
A strategy combining experimental and theoretical approaches enables the investigation of the origins of protein–carbohydrate interactions using the WW domain as the interaction subsystem of the EAS. The strategy involves the chemical incorporation of two guest residues (X and Y) and double-mutant cycle analysis to obtain experimental ΔΔG of glycosylation values (n =  52). Ab initio calculations are used to generate quantum mechanical (QM) descriptors of the interaction subsystem. By correlating these QM descriptors with the experimental ΔΔG values, the electronic origins of protein–N-glycan interactions are uncovered. Adapted with copyright permission from [130].

Examples of rational engineering and systematic design of molecular systems involving carbohydrates are relatively rare. The Engineered Asparagine Stabilizer (EAS) is currently the only portable structural module available for conferring glycosylation-mediated stabilization on proteins, yet its use remains limited. To advance the understanding of N-glycosylation stabilizing effects on proteins at the electronic level, Kelly and co-workers conducted a study utilizing experimental and machine learning techniques (Figure 11), which involveed building a data set with differential folding free energy data for 52 pairs of glycosylated and non-glycosylated proteins [130]. Thermodynamic analysis showed that protein–N-glycan interactions are complex and influenced by various electronic effects. At the electronic level, the study revealed that stabilization results from optimizing electrostatic complementarity, non-polar surface burial, and multiple molecular orbital interactions. Additionally, stereoelectronic effects involving CH–π electron delocalization at the protein–N-glycan interfaces appear to involve weak πC=C→σ*C–H and/or πC=O→σ*Cβ–H interactions [131]. These findings provide valuable guidelines for enhancing molecular force fields used in simulating and designing systems involving protein–carbohydrate interactions.

## 8. Biomedical Applications

Significant efforts have been made to achieve site-specific modification of proteins, with applications ranging from fluorescent probes to photocaging groups, which can elucidate the dynamics and interactions of target proteins in animals and live cells [132,133]. The chemical conjugation is also important for cancer therapies, such as antibody-drug conjugates. The US Food and Drug Administration has approved various therapeutic protein conjugates with PEGs, which offer many advantages, including protein stability and extended circulation half-life [18,134,135]. This section highlights site-selective protein modification towards specific applications.

**Figure 12:**
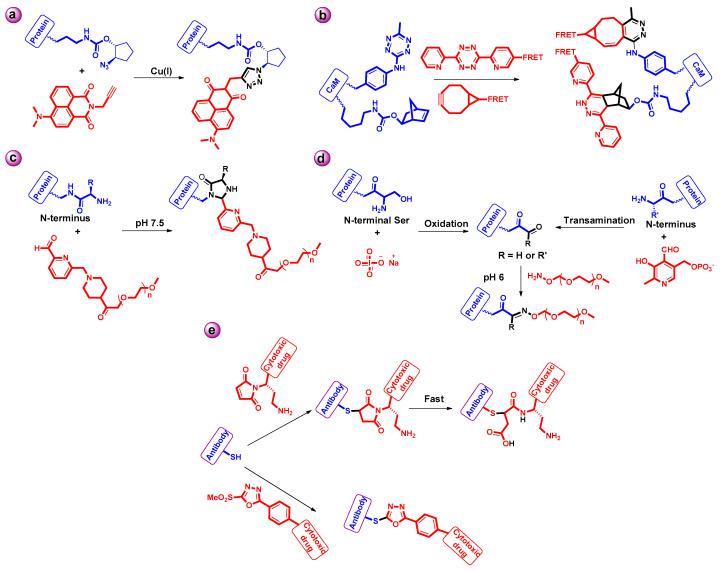
Protein-modification chemistry for cell imaging involves dye based strategies, such as, (**a**) Cu(I)-catalyzed azide–alkyne cycloaddition between 4-N,N-dimethylamino-1,8-naphthalimide (4-DMN) and azide-containing proteins; (**b**) tetrazine can react with a strained cyclooctyne rings or norbornene for labeling; (**c**) PEG based proteins for therapeutics, 2-pyridinecarboxylaldehyde PEG derivatives can be conjugated to native proteins at pH 7.5; (**d**) site-selective PEGylation can also be achieved by introducing N-terminal aldehydes through oxidation or transamination using sodium periodate or pyridoxal phosphate followed by oxime ligation with aminoxy PEG derivatives; (**e**) Antibody–drug conjugates for the delivery of cytotoxic drugs employ chemoselective reactions such as the conjugation between maleimides and thiols resulting in stable ring-opened products, and protein surface Cys reacts with cytotoxic drug-containing reagents, such as methylsulfonylbenzothiazole reagent [3].

In modern biology, biological macromolecules activated by visible light, dyes, or radionuclides are used in diverse imaging applications. The visible-light active biological macromolecules dyes or radionuclides are used in modern biology for diverse imaging applications. While site-selectivity proteins labeling is often necessary for in vitro studies, it is critical for in vivo applications due to its impact on pharmacokinetics and biodistribution [136,137]. Commercially available reagents, such as maleimide, and haloacetamides, are used for protein labeling in purified protein studies. For instance, two distinct native amino acids can be modified with different dyes [138]. Techniques like azide–alkyne cycloaddition, bioorthogonal chemistry, and the Diels–Alder cycloaddition enable the labeling of non-native amino acids (Figure 12a,b) [139,140,141].

Therapeutic proteins have rapidly emerged as a significant class of drugs [142,143]. The conjugation of PEG chains to proteins, known as PEGylation, was first utilized to extend the plasma circulation half-life and reduce immunogenicity in vivo [144]. For example, site-selective PEGylation can be achieved through modifying the N-terminal serine with sodium periodate or transamination and pyridoxal phosphate (Figure 12c,d) [145,146].

Antibody conjugation to proteins for delivering cytotoxic drugs is a widely used approach to enhance the pharmacokinetics and efficacy of antibody–drug conjugates. A common strategy involves modifying the thiol side-chain of genetically engineered cysteine residues through the Michael addition reaction [37,137]. Alternatively, antibody–drug conjugates can be synthesized using Julia–Kocienski-like reagents or by employing the Michael addition of thiol nucleophiles (Figure 12e) [43,147].

## 9. Summary and Outlook

In summary, this review has covered site-specific and target-selective labeling of native proteins, highlighting various chemical approaches. These include modifications at both the N-terminal and C-terminal, as well as targeting specific amino acid residues such as lysine, cysteine, tyrosine, tryptophan, and methionine through chemo- and region-selective pathways. Additionally, affinity-based protein labeling has been discussed, focusing on exchange/cleavage reactions and catalyst tethering techniques. The review also explored linker-directed transfer and ligand-directed chemistry for modifying nucleophilic residue on the protein surface and extended these strategies to affinity-guided catalyst chemistry.

Although numerous methods for site-selective modification exist, no single approach is universally applicable for all scenarios. These techniques have provided molecular insights in various proof-of-concept studies, advancing fundamental biology knowledge. They also have the potential to impact research areas such as cancer and neurodegenerative disorders significantly.

As discussed, a vast toolbox is available for functional protein modification for biomaterials applications. While amine and thiol modification can be achieved through site-selective methods, maintaining structural integrity and functional activity remains a prerequisite. This necessitates the use of orthogonal techniques that combine chemical engineering and protein chemistries to modify the sensitive proteins. Developing new techniques for handling these biomaterials enabled the construction of protein-based functional materials that would otherwise be challenging to create with small molecules. The continuous growth in site-selective protein modification underscores the significant effort in advancing the methods to fabricate the next generation of functional biomaterials. Furthermore, this review also included the application of computational methods, such as molecular dynamics and artificial intelligence, to identify and understand the behavior of amino acids in the bioconjugation process of proteins with small molecules and polymers.

The development of advanced engineering materials requires a combination of multiple modifications and chemical reaction strategies. One major challenge is in predicting the complex structure of proteins following site-selective modifications. Computational techniques have shown promise in accurately estimating the efficacy and screening efforts. Moreover, artificial intelligence has emerged as a powerful tool, particularly in oncology, where artificial intelligence-assisted peptide screening is being used to treat tumors. By integrating these advanced techniques with emerging gene editing tools, such as the CRISPR/Cas9 system, the field of protein and peptide modification could drive the development of materials with specific functions.

## Data Availability

No new data were created or analyzed in this study.
